# Cervical Cancer, Papillomavirus, and miRNA Dysfunction

**DOI:** 10.3389/fmolb.2021.758337

**Published:** 2021-12-10

**Authors:** Evelyn Gabriela Bañuelos-Villegas, María Fernanda Pérez-yPérez, Luis Marat Alvarez-Salas

**Affiliations:** Laboratorio de Terapia Génica, Departamento de Genética y Biología Molecular, Centro de Investigación y de Estudios Avanzados Del I.P.N., México City, Mexico

**Keywords:** cervical cancer, human papillomavirus, HPV, miRNA, microRNA

## Abstract

Cervical cancer is the leading cause of death by cancer in women from developing countries. Persistent infection with high-risk human papillomavirus (HPV) types 16 and 18 is a major risk factor for cervical carcinogenesis. Nevertheless, only a few women with morphologic expression of HPV infection progress into invasive disease suggesting the involvement of other factors in cervical carcinogenesis. MicroRNAs (miRNAs) are conserved small non-coding RNAs that negatively regulate gene expression including genes involved in fundamental biological processes and human cancer. Dysregulation of miRNAs has been widely reported in cervical cancer. This work focuses on reviewing the miRNAs affected during the HPV infection process, as well relevant miRNAs that contribute to the development and maintenance of malignant cervical tumor cells. Finally, we recapitulate on miRNAs that may be used to distinguish between healthy individuals from patients with precancerous lesions or cervical tumors.

## Introduction

In 2020 there were 604,127 new cases and 341,831 deaths of women with cervical cancer worldwide (WHO, 2020). Clinically, cervical tumors can be classified as squamous cell carcinoma (SCC), corresponding to 70–80% of the cases, and adenocarcinoma (AC) with a 10–25% occurrence (Babion et al., 2020a). Other rare cervical tumors exhibit a variety of histological types including malignant adenoma, endometrioid carcinoma, clear cell carcinoma, papillary adenocarcinoma, adenoid cystic carcinoma, adenosquamous carcinoma, and undifferentiated carcinoma, which collectively represent less than 1% of the newly diagnosed cases (Berman and Schiller, 2017; Hull et al., 2020).

Infection with high-risk human papillomaviruses (HR-HPVs) is the primary risk factor for the development of cervical cancer (Castellsagué, 2008; Li and Xu, 2017). Through the expression of two oncogenic viral proteins E6 and E7, HR-HPVs are capable of orchestrating diverse molecular mechanisms that may result in progression to malignant disease. Although persistent infection by HR-HPVs is a necessary cause of cervical cancer, only a few women with morphologic expression of HPV infection progress to invasive disease (Castellsagué, 2008). Hence, other tumor promoter factors must be involved. The identification of such factors is crucial for early diagnosis and could be useful for more efficient treatment and prognosis of cervical cancer.

Most studies generally focus on the genetic dysregulation of host-cell proteins associated with promoting cervical carcinogenesis. In recent years, research turned towards non-coding RNAs (ncRNAs), especially microRNAs (miRNAs). The miRNAs are highly-conserved small double-stranded ncRNAs 19 to 24 nucleotides long that negatively regulate the expression of coding genes through hybridization with complementary or near-complementary sequences within the 3′-UTR of target mRNAs (Felekkis et al., 2010; Bhaskaran and Mohan, 2014). Such binding specifically blocks translation or enables mRNA degradation (He and Hannon, 2004). Because of their functions, miRNAs are essential regulators of many biological pathways associated with cancer development.

### HPV Infection and Cervical Cancer

HPVs are non-enveloped double-stranded DNA (dsDNA) viruses belonging to the *Papillomaviridae* family (Yuan et al., 2021). Most HPVs infect cells from the basal epithelium and have been classified into cutaneous or mucosal types (Burd, 2003). HPV viral particles are relatively small (approximately 60 nm), consisting of an icosahedral capsid containing a single molecule of circular dsDNA of 8,000 base pairs (Harden and Munger, 2017). In general, the HPV genomes encode for six early (E1, E2, E4, E5, E6, and E7) and two late proteins (L1 and L2) (Bansal et al., 2016). There is a third non-coding region in the HPV genome known as the Long Control Region (LCR) or Upstream Regulatory Region (URR) containing the origin of DNA replication as well as transcription control sequences (Harden and Munger, 2017).

The early genes encode for proteins needed for viral replication and transcription. E1 is a DNA helicase essential for the viral replication and E2 works as a regulatory protein modulating the expression of E6 and E7 and regulating viral transcription, replication and genome partitioning (Ryndock and Meyers, 2014). E4 is embedded within the E2 gene and promotes the viral escape from the cornified epithelial layers by attaching to cytokeratin filaments, disrupting their structure (Harden and Munger, 2017). The E5 protein enhances proliferation and may contribute to cancer progression through controlling trafficking of proteins and the vacuolar ATPase in endosomes to modulate epidermal growth factor receptor turnover, thus maintaining a constitutive proliferative signaling (Moody and Laimins, 2010). The E6 and E7 proteins are the principal viral transforming factors. E6 binds and degrades p53 and cellular PDZ proteins and activates telomerase activity thus inhibiting the host cell p53-mediated apoptosis and senescence while E7 binds and degrades the retinoblastoma protein (pRB) leading to continuous cell-cycle rounds contributing to malignant progression by inducing genomic instability and abnormal and sustained host cell proliferation (Harden and Munger, 2017).

The viral late region encodes for the structural proteins L1 and L2. L1 is the major component of the virion capsid produced at the upper layer of the differentiated cervical epithelium during infection and is necessary for binding of the viral primary receptor heparan sulfate (HS). L2 is the minor capsid protein and is involved in the entrance of the viral genome into the host cell (Szymonowicz and Chen, 2020).

There are approximately 30–40 HPV types that infect the anogenital tract (Yuan et al., 2021). Depending on the association with cancer and precursor lesions, the HPV types were grouped in two main classifications: high risk (HR-HPVs) and low risk types (LR-HPVs). HR-HPVs types 16, 18, 31, 33, 35, 39, 45, 51, 52, 56, 58, 59, and 68 are commonly found integrated to the host cell genome in cervical tumor cells. Notably, most cervical tumors contain HPV16 or HPV18 which contribute to ∼70% of invasive cervical carcinoma cases globally (Bhatla and Singhal, 2020). HPV strains 6, 11, 40, 42, 43, 44, 54, 61, and 72 are considered as low-risk HPVs (LR-HPVs), often associated with benign anogenital warts and laryngeal papilloma (Okunade, 2020). It is presently unclear whether HPV types 53, 66, 70, 73 MM9, and 82 MM4 belong to high or low-risk classification.

Remarkably, about 80% of people will get an HPV infection at some point in their lives and usually become infected with HPV shortly after the onset of sexual activity. However, over 90% of those infected will spontaneously clear the infection within 9–12 months suggesting an active participation of the immune system in HPV infection (Szymonowicz and Chen, 2020).

**FIGURE 1:**
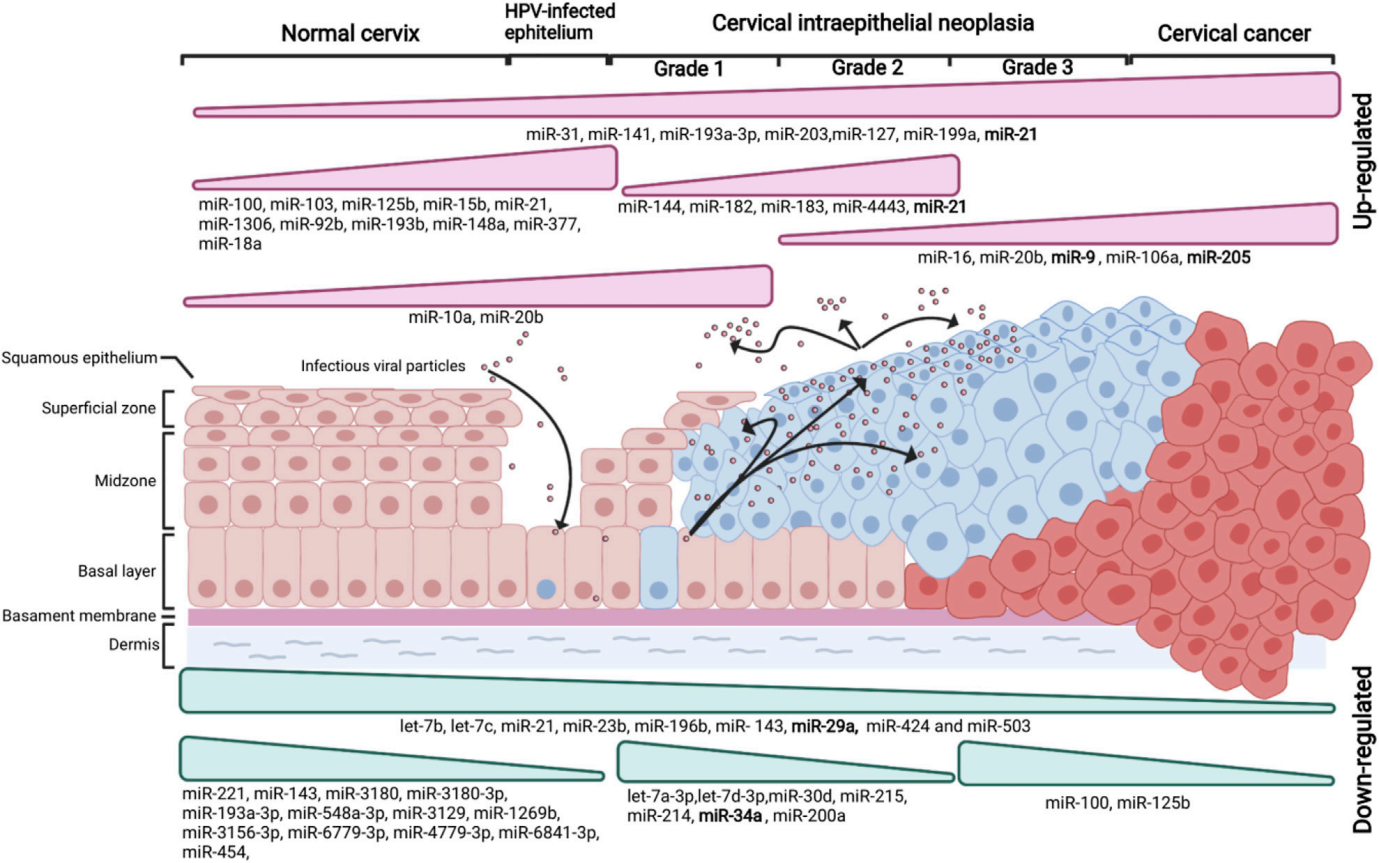
Survey of dysregulated miRNAs in the different stages of cervical carcinogenesis (Created with BioRender.com). Variations in miRNA expression provide a signature of miRNAs in almost every stage of cervical cancer (CC) development. These miRNA signatures might be advantageous in the diagnosis and the monitoring of CC. The normal cervical epithelium morphology is shown in the left followed by intraepithelial lesions (Grade 1, Grade 2, and Grade 3) and CC. The miRNAs essential for the development and maintenance of CC with a diagnostic potential are shown in boldface.

Persistent infection by HR-HPVs can lead to benign Squamous Intraepithelial Lesions (SIL) (Figure 1). These lesions are classified as Low-grade Squamous Intraepithelial Lesions (LSIL) encompassing HPV infection or mild dysplasia also known as CIN1 (cervical intraepithelial neoplasia grade 1) and High-grade Squamous Intraepithelial Lesions (HSIL) encompassing moderate (CIN grade 2) and severe dysplasia (CIN grade 3). In CIN1 the abnormal proliferative cells are present only in the lower one-third of the epithelium. CIN2 and CIN3 are characterized by the expansion of the neoplasia to the lower two-thirds (CIN2) or more (CIN3) of the epithelium. Sometimes, CIN3 involves the full thickness of the epithelium, and it is also known as *in situ* cervical carcinoma (ISCC). HSILs are developed in 10–20% of women with LSILs, and less than 30% of the ISCC patients may progress to cervical tumors when the neoplasia invades into the stroma underneath (Wang et al., 2014; Shen et al., 2020).

Starting with an HPV infection, the transformation of normal cervical tissue to invasive cervical cancer (ICC) is a multistep process that implicates the sustained action of the oncogenic viral proteins E6 and E7 (Wang et al., 2018). Thus, E6 and E7 promote the transformation of normal cervical keratinocytes into pre-malignant and LSIL states and then to an HSIL with a subsequent transformation to ICC.

### miRNAs and Cancer

Most miRNAs are involved in the regulation of fundamental biological processes, such as proliferation (Lenkala et al., 2014), differentiation (Yao, 2016), inflammation (Zhou W. et al., 2018), apoptosis (Taghavipour et al., 2020), cell cycle (Mens and Ghanbari, 2018), and immune response (Chandan et al., 2020). Due to their wide range of functions, miRNA dysregulation has significant consequences in terms of cellular outcomes enhancing the development of a wide range of diseases, including cardiovascular diseases (Zhou S.-S. et al., 2018), neurological disorders (Hussein and Magdy, 2021), autoimmune disorders (Salvi et al., 2019) and cancer (Peng and Croce, 2016).

In the specific case of cancer, it is well established that miRNA dysregulation plays a pivotal role in the initiation, progression, and dissemination of tumorigenesis. The miRNAs are capable of influencing the main features of the carcinogenic process such as continued proliferative capacity, apoptosis resistance, invasion and metastasis induction, increased angiogenesis and the evasion of growth inhibitor signals (Peng and Croce, 2016).

In several tumor types, miRNAs function by targeting transcripts from tumor suppressors or proto-oncogenes (Wang et al., 2008). Thus, miRNAs are subdivided into oncogenic miRNAs (oncomiRs) and tumor suppressor miRNAs (tsmiRs), respectively (Svoronos et al., 2016). OncomiRs are usually highly expressed promoting tumor progression and maintaining the tumor phenotype. On the other hand, the tsmiRs inhibit tumorigenesis by regulating cell proliferation, invasion, promoting apoptosis, and other processes related to cancer development. These tsmiRs are frequently downregulated in most human cancers (Ali Syeda et al., 2020).

The established connection between miRNAs and cancer is deeply associated with aberrant miRNA expression in different cancer types. The miRNA profile in any tumor type can provide various applications such as therapeutic targets or as diagnostic and prognostic markers. However, for the miRNAs to be used for diagnosis or therapy, there is a need for the understanding of their oncogenic or tumor-suppressive roles and how their dysregulation affects tumor progression. OncomiRs may play a meaningful role in cancer treatment through intervening with their expression by silencing or modulation, while the directed overexpression of tsmiRs may exhibit therapeutic effects.

In the specific case of ICC, several OncomiRs have been described, such as miR-499, which promotes cervical tumor progression by enhancing proliferation, migration, invasion, and apoptosis resistance via tumor suppressor SOX6 targeting (Chen Y. et al., 2020). Another example is miR-18a which is involved in the oncogenic transformation of HPV^+^ cervical cells. A study found that HPV E6 and E7 increased the expression of miR-18a (Morgan et al., 2020). This overexpression leads in turn to higher proliferation, migration, and invasion via silencing of tumor-suppressive Hippo pathway STK4 mRNA.

MiR-125 is a well-known tsmiR in cervical cancer. Several authors established that miR-125 inhibited cervical cancer progression by targeting VEGF, thus inhibiting migration and invasion of cervical cancer cells (Fu et al., 2020). Also, miR-375 exerts tumor suppressor effects in cervical tumors via MELK downregulation, thus promoting cell apoptosis while impairing the proliferation, migration, and invasion (Ding et al., 2020).

Some miRNAs can play dual roles as OncomiRs and tsmiRs in cervical carcinogenesis. For example, miR-9-5p is upregulated in the HPV16^+^ CaSki and SiHa tumor cell lines originating from a SCC. In SCC, miR-9-5p plays an oncogenic role by generating downregulation of TWIST1 and CDH1, thus enhancing EMT induction. However, miR-9-5p expression is low in the HPV18^+^ cervical AC cell line HeLa. In this scenario, TWIST is not repressed, so CDH2 activation might be necessary to maintain a malignant phenotype in cervical AC (Babion et al., 2020a).

### HPV and miRNAs

During infection, viruses play a significant role in regulating host gene expression. Part of the gene regulation mediated by viruses is due to changes in miRNA expression triggered by infection (Girardi et al., 2018). Persistent HPV infection induces abnormal miRNA expression (Zheng and Wang, 2011), which could trigger the transformation processes observed from HPV infection to cervical cancer.

Many researchers have studied the miRNA expression profile related to HPV infection and the development of cervical cancer. In 2020 Babion et al. using microarrays determined the miRNA expression profile associated with HPV infection in eight different passages of HPV-transformed keratinocytes representing different stages of cell transformation induced by HPV infection. In total, 106 mature miRNAs were found differentially expressed through the different cell passages. The most representative miRNAs validated by RT-qPCR were miR-15b-5p, miR-100-5p, miR-103a-3p, miR-125b-5p, found upregulated and only miR-221-5p presented downregulation (Babion et al., 2020b).

Another study correlated the severity of the intraepithelial lesions from women infected with HPV-16 to the expression of a set of four miRNAs. The comparison between HPV16^+^ LSIL and cervical cancer patients showed an increased expression of miR-16, miR-21, miR-34a, and miR-143 relative to the HPV-negative group. In the case of HSIL patients, miR-16 and miR-34a expression changes resulted irrelevant compared to the HPV-negative control group. However, miR-21 expression significantly increased while miR-143 decreased compared to the HPV-negative group (Norouzi et al., 2021).

Nunvar et al. identified dysregulated miRNAs exclusive for HPV-dependent SCC. The authors evaluated miRNA expression profiles in a group of HPV^+^ anogenital tumors by next-generation sequencing (NGS). The set included cervical, vulvar, anal, and tonsillar tumors. A variation among tumor types on the total number of dysregulated miRNAs was observed. However, the highest number of miRNAs differentially expressed was detected in cervical HPV^+^ tumors compared to the other SCCs. They also found more downregulated than upregulated miRNAs in HPV^+^ cervical tumors (Nunvar et al., 2021).

In a separate report, Tong et al. identified a subset of miRNAs associated with HPV status. They performed small RNA-seq in cervical tumor lines (2 HPV-negative and 5 HPV^+^) and the exosomes (EXOs) secreted by these cell lines. Specifically, EXOs presented a total of 32 miRNAs differentially expressed in HPV-positive relative to HPV-negative cell lines. The five most commonly upregulated were miR-92b-5p, miR-92b-3p, miR-193a-5p, miR-193b-5p, and miR-1306-5p, while downregulation was reported for miR-548a-3p, miR-1269b, miR-3129-5p, miR-3180-5p, and miR-3180-3p. In tumor cell lines, 70 miRNAs were found differentially expressed in HPV-positive cells including miR-34a-5p, miR-199b-5p, miR-193a-3p, miR-193a-5p, and miR-365b-5p. Meanwhile, miR-431-5p, miR-432-5p, miR-816a-5p, miR-3180-5p, and miR-3180-3p were observed downregulated in HPV^+^ relative to HPV-negative cells. Six miRNAs (miR-146-5p, miR-193a-5p, miR-4661-5p, miR-410-3p, miR-3180-5p and miR-3180-3p) were associated with HPV status in both EXOs and cells (Tong et al., 2020). All these findings suggest a potential miRNA expression deregulation associated with HPV-mediated cervical carcinogenesis.

Dysregulated miRNA expression in HPV infection is widely associated with the viral oncoproteins E5, E6, and E7. Downregulation of miR-454-5p, miR-656-5p miR-3156-3p, miR-4779-3p, miR-6779-3p and miR-6841-3p, was observed by microarrays from HPV-negative HT-3 cells ectopically expressing HPV16 E6 and E7. Further validation with RT-qPCR in HT-3 and C33-A (HPV-negative) cells transfected with HPV16-E6/E7 showed a decrease in miR-3156-3p expression in both cell types. A reduction of miR-3156-3p in HPV16/18-positive cervical tumor samples were also reported. These findings indicate that miR-3156-3p expression is associated with HR-HPV infection and the HR-HPV oncoproteins (Xia et al., 2017).

Silencing of E5, E6, and E7 oncoproteins in the HPV16^+^ cervical tumor lines CaSki and SiHa showed that the loss of the viral oncoproteins upregulates miR-148a-3p. Silencing of only E6 and E7 in SiHa cells showed an increased expression of miR-199b-5p and miR-190a-5p. Overexpression of miR-190a-5p was observed by silencing E5 alone in CaSki. Thus, these three miRNAs might be used as biomarkers for the diagnosis of cervical cancer in HR-HPV-infected patients (Han et al., 2018). Additionally, in a cervical tumor microvesicles (CC-MVs) study, HeLa cells transfected with siRNAs against HPV18 E6/E7 showing increased expression of miR-377. These findings suggested that miR-377 may play a role in E6/E7-mediated oncogenesis (Zhang et al., 2020).

Morgan et al. evaluated the role of HPV16 and 18 E6/E7 oncoproteins in the transformation of HeLa (HPV-18) and CaSki (HPV-16) cells. Upregulation of miR-18a was observed after silencing E6/E7. MiR-18a directly targets the STK4 3′-UTR, a tumor suppressor gene that correlates with the Hippo pathway involved in the hyperproliferative state of the tumor cells (Morgan et al., 2020).

### HPV-Coded miRNAs in Cervical Cancer

There is little evidence that HPV is capable of coding its own viral miRNAs. A bioinformatic study predicted novel putative HPV pre-miRNAs among different HPV types. Surprisingly, HPV16 showed the coding potential for three unique pre-miRNAs HPV16-miR-1, HPV16-miR-2, and HPV16-miR-3 located at E6, E1, and L2 ORFs, respectively. Using the miRTar tool, the putative target genes for HPV16-miR-1 were predicted as ARID5B, ZEB2, THBS1, genes involved in cell motility and migration, and STAT5B related to cell adhesion. For HPV16-miR-3, the predicted targets were SYNE1, PDE1B, GATA6, and GULP1 associated with cell death. However, the predicted targets for HPV16-miR-2 (AFF3, FRMD7, IGDCC4, MYRIP, NRN1, PMP22, RBPMS) were not associated with cervical cancer progression. With this approach, the authors suggested that viral miRNAs might facilitate the host immune response evasion through advocating the latent phase of the HPV life cycle, thus increasing the risk for cancer development although no experimental evidence of these viral miRNAs has been provided so far (Weng et al., 2017).

Although it appears that viral miRNAs could play a significant role in HPV infection and cervical cancer development, there is a controversy about the existence and possible function of these putative viral miRNAs. Accordingly, further research is required to establish if HPV is indeed capable to express its miRNAs and define their functions for the diagnosis, prognosis, or therapy against cervical cancer.

### Dysregulation of miRNAs in cervical cancer

Most miRNAs are encoded as individual genes or gathered in clusters along with other miRNAs and either expressed from their own promoters or as passenger transcripts within introns (Cai et al., 2009). Canonical miRNA biogenesis is a stringently regulated process starting with a primary transcript (pri-miRNA) produced by RNApolII transcription containing 5′ cap and 3′ polyA tail (Ha and Kim, 2014).The pri-miRNA structures as a defined stem-loop determined by internal base-pairing and is processed in a structure-driven manner within the nucleus by the *Microprocessor* complex, containing the RNAse III-like enzyme *Drosha* and the RNA binding protein DGCR8, that cleaves the base of the stem generating a 2 nt overhang at the 3’ end. The resulting precursor miRNA (pre-miRNA) retains the stem-loop structure and is exported to the cytoplasm by the Exportin 5/Ran-GTP complex (Gregory et al., 2004). There, a cytoplasmic RNAse III-like enzyme, *Dicer*, cuts out the loop leaving a 21 nt double-stranded miRNA molecule. The miRNA is bound by an RNA-induced silencing complex (RISC) composed of TRBP, *Dicer* and AGO2 in humans (Chendrimada et al., 2005) and one of the strands is used as guide sequence to mediate sequence-dependent silencing of complementary target mRNAs (Lai, 2002). Therefore, mutations on the miRNA promoters, sequence, biogenesis regulatory regions or target sites and alterations on the miRNA biogenesis proteins may cause dysregulation, loss of specificity or even silencing of the miRNA function with deleterious consequences for the cell.

Numerous researchers have characterized some of the mechanisms causing miRNA dysregulation and their expression pattern in cervical cancer. Compelling evidence suggests that alterations in miRNA expression might result from genomic variations of miRNA genomic loci, such as genetic deletions, amplifications, or mutations. For example, 45 deregulated miRNAs were identified in advanced SCC cells associated with high expression of Drosha and a gain of chromosome 5p. These 45 miRNAs include miR-31, miR-141, miR-203 upregulated, and miR-193a-3p downregulated in clinical samples. Additionally, a majority of miRNAs upregulated were identified in Drosha-overexpressing cells, with only five being down-regulated (Muralidhar et al., 2011).

It is well-known that alteration of transcriptional activators and repressors and abnormal DNA methylation modification of miRNA genomic loci often causes an aberrant miRNA expression profile in cancer (Hussen et al., 2021). In addition to the genomic alterations and patterns of methylation of the miRNA gene sequence, dysregulation of the proteins implicated the miRNA biogenesis contributes to the alterations of miRNA expression in cervical cancer (Ali Syeda et al., 2020).

Another factor that modifies miRNA expression in cervical tumors is single nucleotide polymorphisms (SNPs). SNPs within miRNA genes alter their maturation process by destabilizing the miRNA precursor secondary structure, interfering with Drosha and Dicer processing and strand choice (Wang Y. et al., 2021). Therefore, SNPs in miRNAs deregulate their expression, thus affecting the binding to their target genes. These alterations are well associated with cancer development. Zhi, et al. explored the association between the SNPs in miR-21, miR-26b, miR-221/222, and miR-126 in healthy, CIN, and CC patients. The authors showed that rs1292037 in miR-21 might be involved in the development of CIN or CC. In the case of miR-126, they found that rs2297538 and rs2297537 SNPs might be implicated in the progression of CIN to cervical carcinoma. Finally, the SNP rs2745709 in miR-221/222 might be associated with the development of a CIN lesion (Yang et al., 2021).

Furthermore, a case-control study in the Chinese population evaluated the influence of SNPs in the promoter of the miR-17-92 cluster (miR-17, miR-18a, miR19a, miR-19b, miR-20a, and miR-92-1) in the development of SCC. The authors found a lower level of oncogene miR-20a in patients with the rs9588884 GG genotype. Since miR-20a promotes the proliferation, migration, invasion, and suppression of apoptosis of cervical carcinoma cells, the rs9588884 GG genotype seemingly reduces the risk of SCC (Huang et al., 2020). Another case-control study carried out by the same authors found that miR-34b/c rs4938723 C/T polymorphism is also associated with cervical cancer risk in the Chinese population (Yuan et al., 2016). Liu et al. associated the downregulation of let-7i with a higher risk of cancer development by the presence of SNPs in the let-7i promoter (Liu and Ni, 2018).

Infection with HR-HPV causes alterations in miRNA expression which are essential to maintain a transformed phenotype and subsequent progress to invasive carcinoma. Different studies evaluated miRNAs expression in a range of cell lines, biopsies, cervical mucus, exfoliated cervical cells, and serum from women diagnosed with SCC. These studies showed highly variable miRNA expression during the different stages of cervical cancer**.**

Lui et al. by direct sequencing found downregulation of miR-196b, miR-23b, miR-143, let-7b, let-7c, and an upregulation of miR-21 in cervical carcinoma cells compared with non-tumorigenic cervical samples (Lui et al., 2007). In a different report, miR-127 and miR-199a were found upregulated in invasive SCC compared with non-tumorigenic cervical squamous epithelial samples. The upregulation of miR-127 was associated with lymph node metastasis. Likewise, miR-199 promoted tumorigenesis by enhancing cell growth (Lee et al., 2008).

Variations in miRNA expression also have been reported during the transition from LSIL to HSIL and invasive CC. Zheng et al. described eight differentially secreted exosomal miRNAs; miR-30d-5p, let-7a-3p, let-7d-3p, miR-215-5p downregulated, and miR-144-5p, miR-182-5p, miR-183-5p, miR-4443 upregulated between patients presenting HSILs from those with LSILs. The same study identified miR-30d-5p and let-7d-3p as strong predictors for clinical diagnosis due to the highly differential expression between patients with CIN1 and CIN2 (Zheng et al., 2019). Moreover, Wang et al. found that overexpression of miR-21 and downregulation of miR-214, miR-34a, and miR-200a expression in plasma is associated with the severity of the cervical lesion. Expression of these miRNAs in HSILs was significantly different from those patients with lower-grade lesions (Wang et al., 2019).

Using a bioinformatics approach, Dai et al. identified the downregulation of KFL4 and ESR1 closely related to poor prognosis triggered by the high expression of miR-21 and miR-16 in CC (Dai et al., 2019). Likewise, a systematic study by Pardini et al. identified miR-29a downregulation and miR-21 upregulation. The same study found miR-10a, miR-9, miR-20b, miR-106, and miR-16 as frequently CC dysregulated miRNAs (Pardini et al., 2018).

Downregulation of miR-188 and upregulation of miR-223 is linked with short survival of cervical cancer patients, while downregulation of miR-125b and miR-99a was associated with the 5-years survival rate (Gao et al., 2018). Qi et al. by bioinformatics assays validated the signature of a set of miRNAs correlated to cancer prognosis (miR-585-5p, miR-216b-5p, and miR-7641) and validate these miRNAs through *in vitro* assays (Qi et al., 2020). Moreover, it was established that miR-503 and miR-424 are expressed as a cluster and it was downregulated in CC cells. Upregulation of miR-424/miR-503 impaired cell proliferation, epithelial-mesenchymal transition (EMT), migration, and invasiveness, but improve autophagy (Chen X. et al., 2020).

Tepe et al., explore the potential diagnosis and prognosis of miRNAs involved in autophagy. To achieve this goal, they evaluate the expression of such miRNAs in SCC, founding miR-30e-5p, miR-30a-5p and miR-30c-5p, miR-143-5p, miR-372-5p, miR-375-5p, miR-520e-5p downregulated in the SCC samples compared with HSILs, whereas miR-96-5p, miR-17-5p, miR-130a-5p and miR-520b-5p were upregulated. Finally, they identified miR-30a-5p, miR-520e-5p, miR-548c-5p and miR-372-5p as significant diagnostics markers for SCC, due to the significant association of this miRNAs with the overall survival of SCC patients (Bayramoglu Tepe et al., 2021).

### MiRNAs, in development, progression, and maintenance of cervical cancer

As shown above, miRNA dysregulation may play a significant role in the onset and progression of cervical cancer. Nevertheless, every study appears to suggest different miRNAs associated with the initiation and progression of cervical cancer (Table 1). For clarity, we enlisted the most frequently dysregulated miRNAs in the different stages of cervical cancer progression and their effect on cellular pathways (Table 2).

**TABLE 1:** Differential expression of miRNAs in cervical carcinogenesis. CIN, cervical intraepithelial neoplasia; SCC, squamous cervical carcinoma.

	Differential expression	miRNAs	References
**HPV infection**			
**VPH infected cells vs normal cervical cells**	Upregulated	miR-100-5p, miR-103a-3p, miR-125b-5p, miR-15b-5p, miR-143-5p, miR-1306-5p, miR-92b-5p, miR-92b-3p, miR-193a-5p, and miR-193b-5p	Babion et al. (2020b); Norouzi et al. (2021); Tong et al. (2020)
	Downregulated	miR-221-5p, miR-3180-5p, miR-3180-3p, miR-548a-3p, miR-3129-5p and miR-1269b, miR-432-5p, miR-816a-5p, miR-431-5p, miR-3156-3p, miR-148a-3p	Babion et al. (2020b); Tong et al. (2020); Xia et al. (2017); Zhang et al. (2020)
**Cervical intraepithelial neoplasia**			
**CIN2/3 vs CIN1**	Upregulated	miR-21, miR-144-5p, miR-182-5p, miR-183-5p, miR-4443, miR-10a	Wang et al. (2019); Pardini et al. (2018); Norouzi et al. (2021)
	Downregulated	let-7a-3p, let-7d-3p, miR-30d-5p, miR-215-5p, miR-214, miR-34a, miR-200a, miR-143	Zheng et al. (2019); (Wang et al. (2019); (Norouzi et al. (2021)
**CIN2/3 vs normal samples**	Upregulated	miR-125a, miR-21	Wang et al. (2019); Bayramoglu Tepe et al. (2021)
	Downregulated	miR-30e, miR-96, miR-30a, miR-130a, miR-302a, miR-143, miR-372, miR-17, miR-375, miR-30c, miR-520e, miR-548c, miR-373, miR-214, miR-34a and miR-200a	Wang et al. (2019); Bayramoglu Tepe et al. (2021)
**Cervical cancer**			
**SCC vs normal cervical samples**	Upregulated	miR-21, miR-127 and miR-199a	Lui et al. (2007); Lee et al., (2008)
	Downregulated	let-7b, let-7c, miR-23b, miR-196b, miR-143	Lui et al. (2007)
**SCC vs CIN1**	Upregulated	miR-96-5p, miR-17-5p, miR-130a-5p and miR-520b-5p miR-16-5p, miR-20b-5p, miR-9-5p, miR-106a-5p, miR-205-5p	Bayramoglu Tepe et al. (2021); Pardini et al. (2018); Norouzi et al. (2021)
	Dowregulated	miR-30e-5p, miR-30a-5p and miR-30c-5p, miR-143-5p, miR-372-5p, miR-375-5p, miR-520e-5p	Bayramoglu Tepe et al. (2021); Norouzi et al. (2021)

**TABLE 2:** Relevant target genes for dysregulated miRNAs in cervical cancer.

miRNA	Clasification	Target	Pathway	Associated events	Reference
miR-9-5p	OncomiR	CDH1	Angiogenesis	Down-regulation of CDH1 leads to activation of β-catenin, resulting in the up-regulation of VEGFA, a proangiogenic factor	Farzanehpour et al. (2019)
miR-9-5p	OncomiR	TWIST1	Metastasis	Suppression of epithelial protein CDH1 and transcriptional activation of mesenchymal marker CDH2	Babion et al. (2020a)
miR-9-5p	OncomiR	SOC5/	Angiogenesis/Radiosensitivity	Increasing EMT transition and tube formation	Wei et al. (2019)
miR-21-5p	OncomiR	PDCD4	Immune evasion	Down-regulation of PDC4 resulting in suppression of the inflammation process via NF-kB activating the anti-inflammatory cytokine interleukin 10	Asangani et al. (2008)
miR-21-5p	OncomiR	PTEN	Tumorigenesis	PTEN down-regulation amplifies PI3K signaling resulting in sustaining proliferative signaling	Bumrungthai et al. (2015)
miR-34a-5p	TsmiR	WNT1	Proliferation/Invasion	Induction of an E-P cadherin switch via the WNT1/β-catenin pathway	(Li et al. (2020))
miR-34c-3p	TsmiR	MAP2	Proliferation	Microtubule stabilization of MAP2 leads to proliferation inhibition and cell death in tumor cells	Córdova-Rivas et al. (2019)
miR-29a	TsmiR	STIR1	Migration and invasion	SIRT1 suppressed E-cadherin expression and promoted N-cadherin expression	Nan et al. (2019)
miR-29a	TsmiR	HSP47	Metastasis	Overexpression of molecular chaperone HSP47 leads to cell migration and invasion	(Yamamoto et al. (2013))
miR-16-1	OncomiR	CCNE1	Cell cycle	Cyclin E1 (CCNE1) promotes transition of cells from G1 to S phase	Zubillaga-Guerrero et al. (2015)
miR-148b	TsmiR	CASP3	Apoptosis	Caspase-3 activated death protease, catalyzing the specific cleavage of many key cellular proteins	Mou et al. (2016)
miR-182	TsmiR	DNMT3a	Apoptosis	Hypermethylation by DNMT3a resulted in the silencing of tumor suppressor genes	Sun et al. (2015)
miR-155-5p	OncomiR	TP53INP1	Invasion	TP53INP1 blocks tumor progression via p53-dependant and -independent pathways	Li et al. (2019a)
miR-452b-5p	OncomiR	WTX, β-Catenin	Invasion/Migration	WTX, β-catenin, β-TrCP2, APC and AXIN1 form a complex that could inhibit cancer progression by ubiquitination of β-catenin protein	Li et al. (2019c)
miR-204	TsmiR	ATF2	Autophagy	Phosphorylated ATF2 bind to promoter region of genes involved in cell cycle and autophagy	Li et al. (2019b)
Let-7a	TsmiR	PKM2	Invasion/Migration	PKM2 in the role of protein kinase contribute to development of tumorigenesis	Guo et al. (2017)

### MiR-9-5p

In humans, miR-9 is coded by three different genes (miR-9-1, miR-9-2, and miR-9-3) located at different chromosomes: 1q22, 5q14.3, and 15q26.1, respectively. The presence of miR-9 sequences in three different loci coupled with the high level of sequence conservation, suggests an important role of this miRNA on the cell cycle and the development of cancer (Nowek et al., 2018).

Expression of miR-9 is upregulated in HPV16-infected LSIL compared with the HPV- negative healthy population, while is significantly reduced in HPV52 and HPV58-infected dysplasia (Liu et al., 2020). These differences suggest that different HPVs may have different infection patterns, and thus the levels of key miRNAs may be differentially affected. This situation reflects the challenges faced by investigations aimed to find differences in the expression levels of miRNAs in cervical cancer, as the natural history of HPV infection is not yet fully understood and it is suspected that this may vary depending on the viral subtype, and that it is practically impossible to determine when the infection has started on a patient tested positive for HPV and how long the infection has been ongoing at the time of sampling (Woodman et al., 2007). Once normal tissue has progressed to HPV-associated dysplasia, it has been observed downregulation of miR-9, resulting in high levels of E-cadherin what leads to activation of β-catenin. The final result is the up-regulation of VEGFA, a proangiogenic factor (Pardini et al., 2018; Farzanehpour et al., 2019). Nevertheless, Babion et al. explored in more detail the role of miR-9 in the most common CC types (SCC and AC), finding that miR-9 expression is influenced by tumor histotype and by HR-HPV type. They found that miR-9-5p could be acting like an oncomiR, by targeting TWIST1 and CDH1. In contrast, miR-9-5p acts as tsmiR in AC, since the EMT phenotype is achieved by low levels of miR-9-5p, which facilitated the upregulation of CDH2 via TWIST1 (Babion et al., 2020a).

### MiR-21-5p

The locus for pri-miR-21 is found within the intronic region of the VMP1 gene located in chromosome 17 (Kumarswamy et al., 2011). MiR-21 is abundantly expressed in mammalian cells and its up-regulation is associated with many cancers.

Lui et al. reported the up-regulation of miR-21 expression in HPV-positive CC compared with that in LSILs, especially in HPV16-infected tumors. Conversely, in cervical tumors infected with HPV52 and HPV58, the miR-21 levels are not statistically different compared with LSILs infected with the same type. These results indicate that miR-21 dysregulation could be useful as a biomarker particularly in HPV16-infected tumors (Liu et al., 2020). Mir-21 targets several genes associated with cancer such as PTEN, PDCD4, RECK, and STAT3 (Asangani et al., 2008; Bumrungthai et al., 2015; Bautista-Sánchez et al., 2020). The role of miR-21 in CC was deepened by Bumrungthai et al. where miR-21 expression in HPV-negative normal cytology was significantly lower than in HPV-positive samples in abnormal tissue and SCC. This result demonstrates that induction of miR-21 might be involved in HPV infection, and cervicitis (Bumrungthai et al., 2015). It has been proposed that the miR-21 overexpression could lead to the down-regulation of PDC4 resulting in suppression of the inflammation process via NF-kB activating the anti-inflammatory cytokine interleukin 10, leading to immune evasion and cervical cancer progression (Asangani et al., 2008). MiR-21 can also control the PI3K/AKT/mTOR pathway by inhibiting AKT activation increasing NF-kB activity leading to inflammation.

### MiR-34a and miR-34c

The miR-34 family consists of miR-34a, located on chromosome 1p36, miR-34b, and miR-34c co-transcribed from chromosome 11q23 (Navarro and Lieberman, 2015). MiR-34a is a classical tumor suppressor gene and is frequently down-regulated in gastric cancer, liver cancer, prostate cancer, and cervical cancer (Zhang et al., 2016). The study of miR-34a in cervical cancer began in 2009 when Wang et al. showed that cervical tumors and cervical cancer-derived cell lines containing oncogenic HPVs displayed reduced expression of tumor-suppressive miR-34a, attributed to the expression of the viral protein E6, which destabilizes the tumor suppressor p53, a known miR-34a transactivator (Wang et al., 2009). Later, Li et al. observed a reduced pri-miR-34a expression in cervical tumors and precancerous lesions. Such diminution could be detected in a very early stage, suggesting that miR-34a inhibition is induced by HR-HPV E6 oncoprotein through the p53 pathway, constitute an early-onset event in the development of CC (Li et al., 2010). The mechanism that has been proposed for miR-34 to promote the proliferation and invasion of cervical cells with SCC is through its target WNT1 by induction of an E-P cadherin switch via the WNT1/β-catenin pathway (Li et al., 2020).

miR-34c is another miR-34 family member it’s been associated with CC. Lopez et al. found an inhibitory proliferation effect of miR-34c-3p and miR-34c-5p in CC SiHa cells. Further, the authors establish that only miR-34c-3p is involved in, apoptosis, cell migration, and invasion. Thus, suggesting that miR-34c-3p and miR-34c-5p may target different mRNAs and thus harbor dissimilar functional roles (López and Alvarez-Salas, 2011). These results were complemented by Rivas et al., who demonstrated that miR-34c-3p mimic transfection led to the clear downregulation of MAP2 protein, as well as of MMP9 activity (Córdova-Rivas et al., 2019). Also found that 5p and 3p strands of miR-34 family members have differential effects in cell proliferation, migration, and invasion in cervical cancer cells, however, the mRNA targets regulated by 5p and 3p arms of miR-34 family members are needed to clarify, and this could elucidate the differential effects of this miRNAs on cell processes.

### MiR-29a

The miR-29 family consists of four members (miR-29a, miR-129b-1, miR-29b-2, and miR-29c) encoded in two gene clusters. In humans, miR-29b-1 and miR-29a are located at chromosome 7, and miR-29b-2 and miR-29c are situated on chromosome 1 (Wang et al., 2015). It has been shown that miR-29a is down-regulated in tumors and HPV-positive LSILs (Yamamoto et al., 2013; Jia et al., 2015; Servín-González et al., 2015). In 2019, Zamani et al. reported a significant decrease in miR-29 in tumor samples relative to the control groups, suggesting that miR-29a could operate as a tumor-suppressor in CC progression (Sara et al., 2019). MiR-29a participated in the migration, invasion, and EMT directly targeting the 3′-UTR of SIRT1 mRNA (Nan et al., 2019). Recently it has been demonstrated that miR-29a regulates the p16 methylation pattern in CC by downregulating DNMT3A and DNMT3B (Wang A. et al., 2021). Also, it has been found that miR-29a regulates HSP47 having a key role in the maturation of collagen molecules. The diminution of HSP47 inhibited cancer cell migration and invasion, suggesting that the miR-29a-HSP47 pathway contributes to the metastasis of cervical SCC (Yamamoto et al., 2013).

### Role of miRNAs as biomarkers in early stages of cervical cancer

Currently, histopathology and cytology (Pap-smear) are the gold standard for detection of HPV-associated dysplasia and cervical cancer. Although efficient and low-cost, these methods have a relatively low sensitivity (about 50%) and heavily rely on interpretation, sample recollection and technician training (WHO, 2014). Recent updates in cervical cancer screening guidelines include the addition of HPV testing to cervical cytology, which provides 60–70% greater protection against ICC compared to Pap-smear alone (Bedell et al., 2020). Nevertheless, more sensitive approaches are needed for early and opportune diagnosis. In this regard, liquid biopsy-based approach may theoretically represent a valid additional (or alternative) model for CC screening, diagnosis, and follow-up (Palmirotta et al., 2018). In liquid biopsy, smalls non-coding RNAs gain a central role in cervical cancer diagnosis and prognosis, being major components in circulating RNA detection and exosome identification (Table 3). The need for markers that indicate the presence of cervical dysplasia in early stages is urgently required, as early stages of cervical carcinogenesis are usually asymptomatic, and in advanced cervical carcinoma the symptoms are general and shared with several conditions (Jia et al., 2015).

**TABLE 3:** Molecular models for early cervical cancer diagnosis and screening.

Method	Description	Role in cervical cancer diagnosis and prognosis	Reference
Circulating tumor cells (CTCs)	Isolation of tumor cells in bloodstream utilizing their physical differences compared with leukocytes	Identification and quantification of HPV oncogenes and epithelial markers, by using molecular and/or immunofluorescence procedures	Palmirotta et al. (2018)
Circulating Cell-Free DNA (ctDNA)	Detection of tumor DNA free in the circulatory system by extremely sensitive detection methods	Finding of distinctive mutated genes in cervical cancer or viral DNA by NGS panels or dPCR.	Palmirotta et al. (2018)
Cell-Free Circulating non-coding RNA	Detection of tumoral ncRNAs in the bloodstream, active release by cancer cells	Searching for lncRNA or miRNA characteristic of CC taking advantage of stability of ncRNAs in plasma compared with other nucleic acid	Cafforio et al. (2021)
Exosomal miRNAs	Analysis of miRNAs shuttled in extracellular vesicles that can be easily detected in body fluids thanks to their abundance and stability	Identify signature DEmiRs in patients with CINs and CC.	Cafforio et al. (2021)
Detection of aberrant methylation pattern	Analysis of methylation pattern in cervical scraps or biopsy in genes	Detection of aberrant DNA methylation of oncogenes and ts-genes using affinity capture of methylated DNA.	Yang et al. (2020)

Because miRNAs are highly stable ncRNA species compared with mRNAs and some IncRNAs and have a relatively high average half-life (5 days on average), miRNAs can be considered as prognostic biomarkers of cervical cancer (Jiang et al., 2020). The potential clinical relevance of certain miRNAs in cervical tissue samples was explored by Park et al., demonstrating the potential to miR-21 and miR-155 combined with an HPV E6/E7 mRNA assay as biomarkers for diagnosis and management of both HPV positive and HPV negative LSILs and cervical tumors (Park et al., 2017). In a study conducted in Chinese population, miR-21 was notably upregulated and suggested as a biomarker to distinguish cervical tumors from LSILs. Another miRNA involved in the differentiation between LSILs and CC is miR-34a, especially in HPV16 infected patients. (Liu et al., 2020).

Recently, Zamanni et al. explored the differences between the levels of miR-21 and miR-29a in HPV^+^ and cervical tumor groups using liquid-based cytology samples (LBCs) in Iranian women. Both miR-21 and miR-29a can serve for cervical cancer diagnosis because of the existing correlation between miR-21 upregulation and the downregulation of miR-29a in tumor samples (Sara et al., 2020). Another study using LBCs showed the up-regulation of miR-205 in HSILs, suggesting the utility of miR-205 expression as a novel triage marker to supplement HR-HPV testing in patients with LSILs (Xie et al., 2017). Analyzing the levels of miR-205 can serve as a supplement to the screening of HR-HPVs, which can help determine which patients with LSILs will progress to HSILs.

Circulating miRNAs are attractive as biomarkers in cervical cancer because this eliminates the need to take solid tissue samples, simplifying sample collection. In this regard, You et al. analyzed three plasma miRNAs (miR-127, miR-205, and miR-218) for cervical cancer detection. The results showed that miR-205 had a higher predictive value with an AUC of 0.843, a sensitivity of 72.00%, and a specificity of 82.35% (You et al., 2015). Comparing these results with those obtained by Xie et al., it is likely that miR-205 could serve as a biomarker both in plasma and in samples from cervical tissue, although further research would be necessary to examine the levels of miR-205 in both sample types.

In another study, Farzanehpour et al. monitored the levels of miR-9, miR-192, and miR-205 in serum and tissue of cervical cancer and LSIL patients infected by HPV in comparison with normal tissue showing an increased expression level of miR-192 in tumor and L-SIL tissues, concluding that miR-192 can be used as a potential biomarker for the early detection of CC (Farzanehpour et al., 2019).

Finally, several research groups focused in establishing a miRNA profile for the detection of cervical cancer. For example, Jia et al. described a panel of 5 serum miRNAs (miR-21, miR-29a, miR-25, miR-200a, and miR-486-5p) as a cervical cancer biomarker. The ROC curves indicated that this panel may constitute a useful fingerprint test for early diagnosis. Interestingly, miR-29a and miR-200a may indicate tumor histological grade and progression stage (Jia et al., 2015).

## Discussion

The impact of miRNAs in cancer has been widely studied because of their capacity to influence many tumorigenic processes, such as sustained proliferative capacity, apoptosis resistance, invasion and metastasis induction and increased angiogenesis. Thus, several studies have focused on the expression profile of miRNAs in different types of cancer and their functions in the tumorigenic process.

In this review, we highlighted the expression and function of four extensively studied miRNAs. Mainly, miR-21 was found up-regulated in HPV infection and through regulation of several target genes is capable of enhancing progression to cervical cancer. Because of the well-established overexpression in cervical tumor tissue, miR-21 is described as a good diagnostic and prognostic marker. Also, miR-9a has been found upregulated in SCC associated with EMT through regulation of TWIST1 and CDH1 contributing to invasive CC development. In the case of miR-34a and miR-29, they are both downregulated in cervical carcinogenesis and are well known for their tumor suppressor functions. These miRNAs are associated with an ICC state through the regulation of genes involved in migration, invasion, and EMT (WNT1, SIRT1, and HSP47).

Due to the discovery of miRNA aberrant expression associated with cervical cancer, the new molecular mechanism of cervical tumorigenesis has emerged providing opportunities for miRNAs become useful for clinical applications. Accordingly, it is important to categorize the expression and the function of miRNAs in the different stages that lead to cervical cancer (Figure 1); from the infection with HR-HPV, followed by subsequent LSIL to malignant transformation. Aberrant miRNA expression begins with HPV infection and despite most infections are spontaneously cleared in many patients, the HPV infection persists. Consequently, the expression profile of miRNAs continues changing throughout the cervical carcinogenesis development process. As mentioned above, the upregulation of miR-205 could be a marker for the diagnosis of HPV^+^ intraepithelial lesions. The use of this miRNA could be more accurate than the determination of the HR-HPV oncoproteins in cervical tissue. In invasive SCC the overexpression of miR-21 and downregulation of miR-29 stands out in tumors compared to normal cervical tissue providing a molecular signature useful for cervical cancer diagnosis.

Most miRNAs possess some advantages that could turn them into ideal candidates as biomarkers for the diagnosis and prognosis of cervical cancer. For example, their stability, accessibility for measurement, specificity for the tissue or cell type and a putative capability to be more informative than other biomolecules. Although these advantages suggest a promising clinical implication of miRNA-based diagnosis the validation of miRNA as biomarkers has not been successful. This can be explained by many methodological challenges including sample collection, storage, extraction methods, quality controls, differences in methodology of the studies, the lack of standard methods for normalization, and the inability to discriminate among closely related miRNAs (Saliminejad et al., 2019). Besides the potential technical biases mentioned above, other critical variables that could have deep implications in the accurate interpretation of miRNA biomarker studies are related to the intrinsic variability, such as the heterogenicity of the tumor itself since miRNA expression pattern may fluctuate among different patients with the same type of cancer due to the individual variability such as race and gender, life-style and external factors, drugs, smoking habits, and other conditions (Tiberio et al., 2015).

Thus, considering all these limitations, every miRNA-based diagnosis study requires further steps of validation and a proper standardization of all analytical procedures, to control for all potential technical biases. Furthermore, deeper studies with a larger number of patients enrolled are required to discover a signature of specific and sensitivity miRNAs capable of discriminate between HPV-infection, dysplasia and cancer patients from healthy subjects.

In addition, the individual variability between different cervical cancer patients is not negligible and can influence miRNA application in clinical practice. Accordingly, miRNAs could be used as a complementary but not definitive diagnostic tool requiring the input from standard diagnosis practices such as Pap-smear cytology, histopathology, and HPV testing.

Besides diagnosis, some clinical trials have focused on the miRNA therapeutic efficacy in cervical cancer. Potential miRNA-based cervical cancer therapies focus on the delivery of miRNA mimics to restore the functions of tsmiRs or delivering antagomiRs to repress oncomiRs (Hanna et al., 2019). However, there are some challenges to overcome to develop miRNA-based therapies, such as degradation by nucleases upon addition into biological systems, poor delivery to target cells, off-target effects, and activation of immune responses (Segal and Slack, 2020). Despite the off-target effects that miRNA-based therapies may have, they are promising tools due to their many advantages compared with the highly unspecific and toxic therapies currently applied such as chemotherapy and radiotherapy. For example, because the anatomic location of cervical tumors, miRNAs can be easily delivered in the specific tumor site reducing the damage to normal cells.

The clinical implications of miRNA-based diagnostic and therapeutic strategies are necessary to reduce the mortality and successful management of cervical cancer. Thus, further studies are required to elucidate the role of miRNAs in cervical carcinogenesis and the mechanisms through which miRNAs regulate cellular process enhancing tumorigenesis. Those studies might provide suitable evidence of the role of miRNAs as diagnostic and prognosis biomarkers, as well as treatment molecules for cervical cancer.
